# The significant association between maternity waiting homes utilization and perinatal mortality in Africa: systematic review and meta-analysis

**DOI:** 10.1186/s13104-019-4056-z

**Published:** 2019-01-14

**Authors:** Bayu Begashaw Bekele, Tegene Legese Dadi, Thomas Tesfaye

**Affiliations:** 1 Department of Public Health, College of Health Sciences, Mizan Tepi University, Mizan Aman Street, 260, Mizan Aman, Ethiopia; 2 Arba Minch College of Health Sciences, Arba Minch, Ethiopia

**Keywords:** LMICs, Meta-analysis, MWHs, Perinatal mortality, Systematic review

## Abstract

**Objective:**

A proper uptake of maternity waiting homes (MWHs) is important to improve maternal and child health (MCH). The aim of this review is to generate the best existing evidences concerning the MWHs utilization and its impact on perinatal mortality (PNM) among pregnant mothers in Africa. Both relevant quantitative and qualitative studies, investigated and reported from databases were explored. Meta-analysis of the studies was displayed by tables and forest plots. The Stata version 14 was used with the fixed effect model and 95% confidence interval.

**Results:**

In this review, a total of 68,805 births were recorded in this review. About 1.6% and 7.2% PNM occurred among non-exposed and exposed mothers respectively. Fifty percent of the studies showed there is a significant association between MWHs use and PNM. Meta-analysis revealed that utilizing MWHs have a significant effect in a reducing PNM by 82.5% (80.4%–84.5%), I^2^ = 96.5%. Therefore, use of MWHs has a potential to reduce PNM among pregnant mothers. The review revealed that MWHs relevance to achieving sustainable development goals (SDGs) concerning reducing newborn mortality. Therefore, the utilization rate of MWHs must be enhanced to achieve SDGs.

**Electronic supplementary material:**

The online version of this article (10.1186/s13104-019-4056-z) contains supplementary material, which is available to authorized users.

## Introduction

Globally, the women and child health is the fundamental concern not only to the women themselves, but it’s about their newborns, close family and the country as a whole. Thus, maternal health is an important policy’s feature and planning for healthcare as being the focus of SDGs. The actual operationalization of maternal healthcare continuum ensures that mothers receive essential health packages from pre-pregnancy to birth, and postnatal reducing the risk of maternal and perinatal death [1, 2].

Moreover, over a million babies are stillborn per year; among them, at least 300,000 die during labor. A further 1.16 million babies die in their first month of life up to half on the first day. Four million low birth weight babies and others with neonatal complications live and a similar number of African women have non-fatal complications of pregnancy [3]. But these are preventable. For instance, about 800,000 lives could be saved each year if essential interventions already in the policy were reached 90% of African mothers [4].

Hence, to alleviate the aforementioned problems MWHs is a strategy especially for communities who were unable to reach facility easily for delivery and postpartum services [5–8]. It is a facility where pregnant mothers, especially who are from hard to reach areas stay until their date of delivery [9].

Although the strategy has powerful positive outcomes on improving MCH, it seems unfocused [10–13]. According to the African Union report explanation maternal, newborn and child morbidity and mortality are extremely high in Africa. This resulted in a negative burden on the continent’s socio-economic well-being [14]. Because in Africa where financial, technical and geo-cultural barriers to care seeking as well as perceptions of poor quality of services at health facilities diminish the utilization of services [15]. Besides these deficiencies; policies and programs encouraging skilled attendance and institutional delivery are missing the poorest populations, where most mothers deliver at home [16–18]. Despite its vitality, internationally few studies were tried to sketch its importance and association with PNM.

Therefore, this review is important to assess the pooled estimate of global existing MWHs utilization association with PNM. The cumulative findings will help in sustaining the utilization and saving lives of both mothers and newborns.

## Main text

### Methods

Preferred Reporting Items for Systematic Reviews and Meta-Analyses (PRISMA) [19] checklist was used to report the review.

#### Study protocol registration, design, eligibility and search strategy

This review was conducted according to an apriori record has been published on the PROSPERO database [20].

The Cochrane Library (http://www.cochranelibrary.com/), ClinicalTrials.gov/Meta-Registry of trial Registries (http://www.clinicaltrials.org, http://www.controlled-trials.com) and National PROSPERO International prospective register of systematic reviews (http://www.crd.york.ac.uk/prospero) databases were searched. That was an effort to be sure whether systematic review or meta-analysis exists and for the availability of ongoing projects related to the current topic. MEDLINE/PubMed, Cochrane library, SCOPUS, CINAHIL and Directory of Open Access Journal (DOAJ) databases were searched systematically. The Medical Science Heading (MeSH) terms were also used. The search was restricted to the criteria (Additional file 1).

#### Outcomes

Studies reported the number of pregnant women both utilized and non-utilized MWHs and PNM between the groups. According to WHO definition, we considered PNM as stillbirth (fetal death), and early neonatal deaths (first 7 days postpartum) [21].

#### Study selection, quality assessment and data extraction

Two authors independently selected and extracted the data from abovementioned databases and articles using a Microsoft Excel format respectively. In addition, using study design; geographic location; years of study; sample size; average percentage of participants; selection of study participants; outcome definition (specific definition criteria of WHO) were filtered. For identified articles, titles and abstracts were reviewed to retrieve studies on the association between utilization of MWHs and PNM. Articles found truly relevant by title and abstract were taken to full-text review for eligibility (Additional file 2). The quality of eligible studies was assessed using Newcastle–Ottawa quality assessment scale was used [22]. Disagreement among the reviewers was solved by discussion and articles were included after consensus was made. Authors strictly assessed for any serious defects as they can increase the risk of bias. Studies were judged to be at low risk of bias (≥ 50% points) or high risk of bias (< 50% points) (Additional file 3).

#### Data synthesis and analysis

Studies that reported PNM (i.e. stillbirths, early and neonatal deaths) and the utilization rate estimates of MWHs among pregnant mothers were assessed by pooling the study-specific estimates using fixed-effect meta-analyses. All analysis were performed using both Stata version 14 and RevMan version5 [23]. Statistical tests were two-sided and used a significance cut off point of p value of < 0.05. When studies reported point prevalence estimates made at different follow-up period within the stay at MWH, the overall stay period was used. Intuitive index (I^2^) and harbours tests statistic that means the percentage of variability across primary studies. That estimates due to heterogeneity rather than sampling error, or chance, with values 50–90% indicating that substantial heterogeneity). Sensitivity analysis was performed by excluding each study with high and low supremacy over the overall pooled prevalence estimates. Hence, two studies [24, 25] with highest and lowest determinants on pooled estimate (Additional file 4). After exclusion of both studies MA showed the significant association between MWHs utilization and PNM (pooled estimate OR = 0.31 95% CI 0.26–0.37, I^2^ = 89). Results from studies grouped according to pre-specified inclusion criteria were compared using stratified meta-analysis or random-effects meta-regression. Bias secondary to small study effects was investigated using funnel plots.

### Results

#### Study characteristics

239 relevant articles were identified from PubMed/MEDLINE (132), SCOPUS (78), DOAJ (6), Cochrane Library (4) and CINAHL (16) and other sources (3) for further screening. Among 32 eligible full-text articles, ten studies were included for systematic review and meta-analysis Those included studies containing a total of 68,801 births. Four from Ethiopia, two from Zimbabwe [26, 27], one each from Zambia [25], Malawi [28], Liberia [29], and Tanzania [30]. All of the studies were facility-based studies. Six from hospital [24–27, 29, 31] and one from health center level [28]. Two retrospective [24, 32], four cross sectional [30, 33–35], and four were prospective studies [26, 27, 31, 35]. The studies follow up period ranges from 4 months to 22 years. However, there is no independent interventional study measured the effect size or association between two variables.

All included studies had assessed the association between MWH utilization and perinatal mortality. But the way they measured MWH utilization was mentioned (Additional file 5).

#### Meta-analysis of association between MWHs and perinatal mortality

Five or fifty percent of studies [24, 26, 27, 31, 32] showed there is a significant association between MWH utilization and PNM.

Those included studies containing a total of 68,801 childbearing age and pregnant mothers from ten African countries. Among these 21,504 (31.2%) mothers utilized (non-exposed) and 47,301 (68.8%) not utilized MWHs (exposed). The magnitude of MWHs utilization among pregnant women magnitude is 31.2%. Among utilizers of MWHs 314 perinatal deaths occurred and making PMR about 16.7/1000 live births. Among 31,571 controls there were 3855 perinatal deaths making PMR of 122.1 per 1000 live births.

**Fig. 1:**
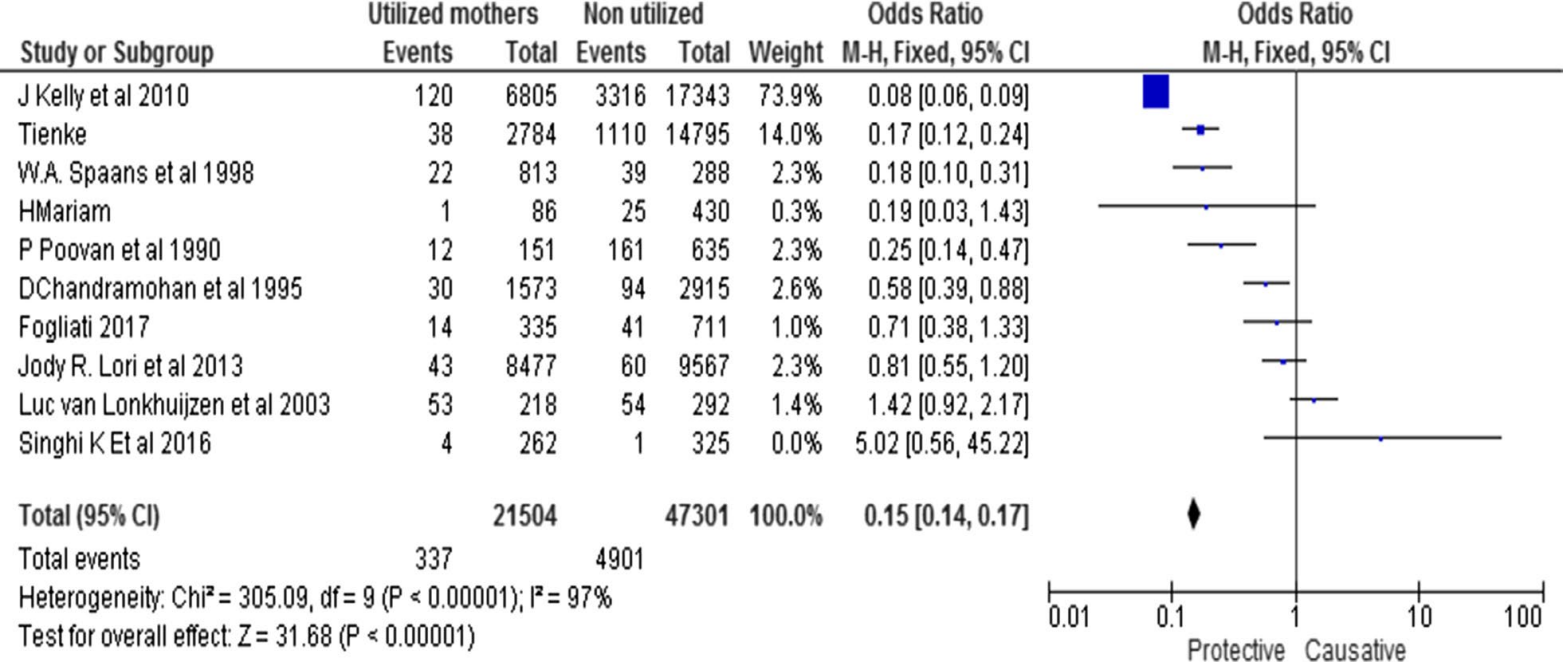
Forest plot of the meta-analysis

In meta-analysis, pregnant women who utilized MWHs are less likely to have PNM than who didn’t utilize (OR [95% CI] = 0.175 [0.155, 0.196], Q = 260.5, p < 0.0001). Heterogeneity test indicated I^2^ = 96.5, although the fixed effect model was assumed in the analysis. In other words, not utilizing MWHs increases PNM by 82.5% (95% CI 82.4%–84.5). Conversely, those mothers who did not use MWH have higher odds of PNM than the utilizers (Fig. 1).

#### Heterogeneity and publication bias

Pooled fixed effect odds ratio (95% confidence interval) of PNM compared among mothers who utilized and non-utilized MWHs. p values were calculated for the heterogeneity test. All ten included studies were assessed for heterogeneity and publication bias. Consequently, the analysis showed a substantial heterogeneity of Q test (p < 0.001) and I^2^ statistics (I^2^ = 88%) after sensitivity analysis was done. Hence the fixed effect model was assumed in the analysis. The funnel plot for publication bias showed asymmetry showing there is the presence of bias. In addition, this was confirmed by harbord tests. Because this test has big power to detect the presence of publication bias than other tests like egger and begg’s tests (Additional file 6). This might have resulted from selective reporting, only African studies included, or few studies included in the study.

#### Sensitivity and subgroup analysis

Sensitivity analysis of the ten studies was done to test the effect of each study on the pooled result by omitting each study step by step (i.e. based on nine studies), hence fixed effect model was assumed in the analysis. The sensitivity test was done, and due to two studies from [24, 25], change was noted on overall estimate.

On the other hand, we did subgroup analysis by study design to minimize heterogeneity across studies. It showed a significant reduction in heterogeneity by study design. As the result, it showed a moderate heterogeneity within cross sectional studies (p = 0.074) and I^2^ statistics (I^2^ = 56.7%) after sensitivity analysis was done.

### Discussion

Our study intent was to investigate pool effect estimate of the association between MWHs and PNM among pregnant mothers in LMICs. Because those countries have been noticed by poor MCH care services; suffering from huge maternal and perinatal mortality. Consequently, the findings revealed that there is a high association between MWHs utilization and PNMalthough the variability across the study was very high. In this review the pooled magnitude of utilization of MWHs is low. This is in line with individual studies from Tanzania, Kenya and Laos [11, 36].

In this study mothers who utilized MWHs have less risk of having perinatal mortality than their counter parts. This is in agreement with findings from Laos, Nicaragua, Peru, Ethiopia and Guatemala [11, 37–40]. In addition, other qualitative synthesis and scoping review concerning MWHs utilization and PNM showed that MWHs have the capacity to reduce newborn mortality [5, 41, 42]. This might be due to the fact that timely risk identification and having skilled delivery at health facilities enhance the health of both mothers and newborns [43]. Furthermore, staying at MWHs prevent all three delays; namely first, second and third delays. According to WHO recommendation pregnant women shall stay at MWHs in the last weeks of her gestational age at nearby health facilities equipped with basic emergency obstetric cares services [44]. Thus, happenings of delays are inevitable if pregnant mothers effectively use MWHs.

This review showed there is substantial heterogeneity. Despite, studies measured the association between exposure and outcome, there was variation across the study. This might have resulted from the variation in study design, income level, publication bias, selective reporting or non-reporting. The other possible speculation is this review included studies only from Africa which might conceal or excluded else studies out of Africa resulted in increased heterogeneity.

The strengths of this review are including studies included and focused on exposure and their study subgroups. The included studies were that never yielded any lost to follow up of the cases. In addition, the most important databases like SCOPUS, CINAHL, PubMed/MEDLINE, DOAJ and Cochrane Library were searched by independent authors and agreement was made among authors on exclusion and inclusion. For eligible abstracts but not freely accessible to get full text articles the frequent communication was made with their corresponding authors and included in the study.

### Conclusion

The utilization of MWH among pregnant women is still low. In addition, increasing the uptake of MWH is a promising strategy to bring the progress made to date in reducing newborn, by ending all such preventable deaths before 2030. Therefore, to achieve SDGs it is recommended for all pregnant women to be admitted to MWHs before delivery.

## Limitations

The potential limitations of our study were as follows. There is moderate heterogeneity in this review. This might have resulted from either statistical or conceptual variations across the studies. However, we did subgroup analysis to decrease heterogeneity. Also, only published in English language researches were included in the analysis. Another shortcoming in our study is both cross sectional and cohort studies were included due to lack of interventional studies. Moreover, most studies selectively reported the relationship between MWHs utilization and PNM.

## Additional files

**Additional file 1:** Inclusion and exclusion criteria of reviewed articles.

**Additional file 2:** Flow chart of study selection.

**Additional file 3:** Quality of included studies according to Newcastle Ottawa Quality Scale Assessment tool.

**Additional file 4:** Sensitivity Analysis.

**Additional file 5:** Included Studies in the systematic Review and Meta analysis.

**Additional file 6:** Funnel plot for meta-analysis of the perinatal mortality publication bias among mothers who utilized MWH compared with non-utilized ones.

## Abbreviations

LMICs: low and middle income countries
MCH: maternal and child health
MWHs: maternity waiting homes
OR: odds ratio
PMR: perinatal mortality rate
PNM: perinatal mortality
SDG: sustainable development goal
WHO: World Health Organization
