# Supplementary material for: The significant association between maternity waiting homes utilization and perinatal mortality in Africa: systematic review and meta-analysis
Source: BMC Res Notes. 2019 Jan 14;12:13. doi: 10.1186/s13104-019-4056-z (PMC6332606; doi:10.1186/s13104-019-4056-z)
Supplement: Supplementary file 1 — Additional file 1: Inclusion and exclusion criteria of reviewed articles. [file 13104_2019_4056_MOESM1_ESM.docx]

Inclusion and exclusion criteria of reviewed articles

| **Inclusion criteria** | **Exclusion criteria** |
| --- | --- |
| - Since 1950s - Original articles - Study design: cross sectional, case-control, cohort studies - Reported MWHs utilization and PNM among pregnant mothers - Peer-reviewed and published in English language | - Any reviews - Ecological studies - Letters, editorials, communications, guidelines, protocols |
