# Supplementary figures and images for: The significant association between maternity waiting homes utilization and perinatal mortality in Africa: systematic review and meta-analysis
Source: BMC Res Notes. 2019 Jan 14;12:13. doi: 10.1186/s13104-019-4056-z (PMC6332606; doi:10.1186/s13104-019-4056-z)

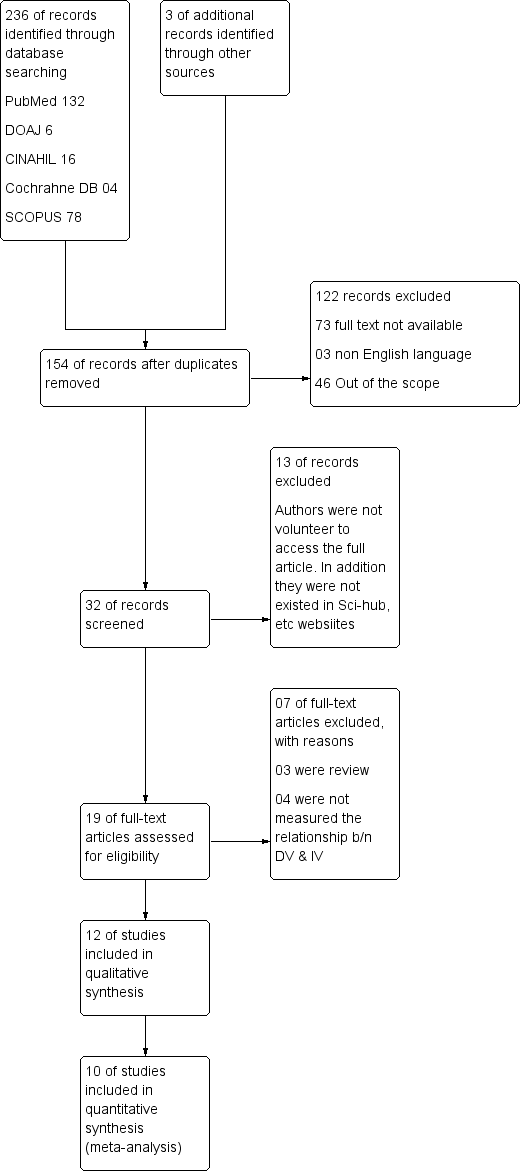


Included

Eligibility

Screening

Identification

Flow chart of study selection

Supplement: Supplementary file 2 — Additional file 2: Flow chart of study selection. [file 13104_2019_4056_MOESM2_ESM.docx]
