# Supplementary material for: The significant association between maternity waiting homes utilization and perinatal mortality in Africa: systematic review and meta-analysis
Source: BMC Res Notes. 2019 Jan 14;12:13. doi: 10.1186/s13104-019-4056-z (PMC6332606; doi:10.1186/s13104-019-4056-z)
Supplement: Supplementary file 3 — Additional file 3: Quality of included studies according to Newcastle Ottawa Quality Scale Assessment tool. [file 13104_2019_4056_MOESM3_ESM.docx]

Quality of included studies according to Newcastle Ottawa Quality Scale Assessment tool

| **Assessment points** | Tienke, et al | H/mariamet al | Singhi et al | Fogliati et al | Jkelly et al | WA Spaans et al | P Poovan et al | Chandaramohan et al | JR Lori et al | LucvanL et al |
| --- | --- | --- | --- | --- | --- | --- | --- | --- | --- | --- |
| Representativeness (2) | 1 | 1 | 2 | 2 | 2 | 1 | 1 | 2 | 2 | 1 |
| Sample size adequacy(1) | 1 | 0 | 1 | 2 | 1 | 1 | 0 | 1 | 1 | 1 |
| Non-response rate(1) | 0 | 1 | 0 | 1 | 0 | 0 | 0 | 0 | 1 | 1 |
| Exposure ascertainment(3) | 2 | 1 | 2 | 2 | 2 | 2 | 2 | 2 | 2 | 2 |
| Comparability(1) | 1 | 1 | 1 | 1 | 1 | 1 | 1 | 1 | 1 | 1 |
| Outcome ascertainment(3) | 2 | 2 | 2 | 2 | 2 | 2 | 2 | 2 | 2 | 2 |
| Statistical test used (1) | 1 | 1 | 1 | 1 | 1 | 1 | 0 | 1 | 1 | 1 |
| Total score | 8 | 7 | 9 | 10 | 9 | 8 | 6 | 9 | 10 | 9 |
| Percent | 66.7 | 58.3 | 75 | 83.3 | 75 | 66.7 | 50 | 75 | 83.3 | 75 |
