# Supplementary material for: The significant association between maternity waiting homes utilization and perinatal mortality in Africa: systematic review and meta-analysis
Source: BMC Res Notes. 2019 Jan 14;12:13. doi: 10.1186/s13104-019-4056-z (PMC6332606; doi:10.1186/s13104-019-4056-z)
Supplement: Supplementary file 4 — Additional file 4: Sensitivity Analysis. [file 13104_2019_4056_MOESM4_ESM.docx]

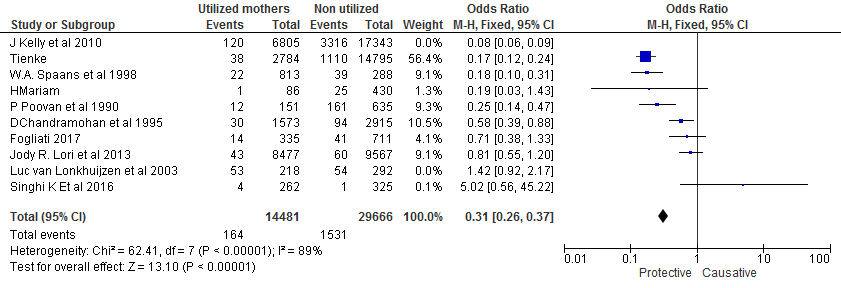
 Abbreviations: CI, confidence interval; df, degrees of freedom; M–H, Mantel–Haenszel

Sensitivity Analysis
