# Supplementary material for: The significant association between maternity waiting homes utilization and perinatal mortality in Africa: systematic review and meta-analysis
Source: BMC Res Notes. 2019 Jan 14;12:13. doi: 10.1186/s13104-019-4056-z (PMC6332606; doi:10.1186/s13104-019-4056-z)
Supplement: Supplementary file 5 — Additional file 5: Included Studies in the systematic Review and Meta analysis. [file 13104_2019_4056_MOESM5_ESM.docx]

| **S.no** | **Study** | **Participants** | **Study design** | **Study country** | **Exposure/Intervention** | **Outcome measurement** | **Main findings** | **Source of funding** | **Quality Score** |
| --- | --- | --- | --- | --- | --- | --- | --- | --- | --- |
|  | J Kelly et al[26] | All women admitted for delivery 1987-2008 | Retrospective | Ethiopia | Maternity waiting area (homes) | PNM measured as stillbirth (the pregnancy outcome resulted in dead baby) | Substantial lower rate of still birth among MWH utilized mothers. Among utilizers and non-utilizers there was 2 and 19 stillbirths per 100 live births. | No specified | 8/14 |
|  | WA Spaans et al[29] | All mothers delivered in hospital from 1 Jan-31 Dec 1991 | Prospective | Zimbabwe | Maternity waiting area (homes) | Stillbirth | Perinatal mortality did not differ significantly OR of 0.6, with 95% CI 0.2-1.4 between hospital 22/1000 and home 39/1000 births. | Not specified | 7/14 |
|  | Poovan et al[35] | All mothers delivered in the Atat hospital from 1987 to 1989 | Prospective | Ethiopia | MWHs | PNM included stillbirth | PNM among non-utilized is 10 times more than utilized ones | Not specified | 9/14 |
|  | Chandramohan et al[30] | All mothers delivered in the Chipinge hospital from 1989 to 1991 | Prospective | Zimbabwe | MWHs | Perinatal mortality | MWHs have capacity to reduce PNM  Most of users were residents from furthest area to MWHs | Not specified | 12/14 |
|  | JR Lori et al[33] | 5 communities with MWH and 5 communities did not (control group) | Prospective cohort | Liberia | MWHs | PNM | The utilization and accessibility of MWH have positive effect in saving lives of neonates and mothers when compared to non-utilization | USAID, 1 K01 TW00 NINR and NIH | 11/14 |
|  | LV Lonkhuijzen et al[31] | All mothers delivered in Nyanje RCZ Hospital from May to November 1994 | Prospective | Zambia | MWHs | PNM was measured as stillbirths | There is no statistical difference between user and non-users of MWHs on newborn mortality but there is no MM among utilizers. | Not specified | 10/14 |
|  | Singhi K et al[32] | Mothers at MWHs at two health centers | Cross sectional | Malawi | MWHs | Stillbirths | The PNM is higher among users than non-users. | BMG Foundation | 8/14 |
|  | Tienke et al, 2018 [37] | Pregnant mothers | Retrospective | Ethiopia | Accessibility of MWHs and utilization | Stillbirths | Higher PNM among non-utilizers. | VSO | 10/14 |
|  | Hailemariam et al 2017[39] | Pregnant mothers | Cross sectional | Ethiopia | MWHs | PNM measured Intrauterine Fetal Death(stillbirth) | Higher PNM among non-utilizers. Only one stillbirth occurred among utilizers and 25 PNM were recorded among non-utilizers. | Hawassa University | 7/14 |
|  | Fogliat[41] | Pregnant mothers | Cross sectional | Tanzania | MWHs | PNM (considered early neonatal deaths and stillbirths) | There is significant association between MWH utilization and PNM. Mothers who didn’t utilized MWHs were lost their PN 5 times than their counter parts. | Not specified | 9/14 |

Included Studies in the systematic Review and Meta analysis
