# Supplementary material for: The significant association between maternity waiting homes utilization and perinatal mortality in Africa: systematic review and meta-analysis
Source: BMC Res Notes. 2019 Jan 14;12:13. doi: 10.1186/s13104-019-4056-z (PMC6332606; doi:10.1186/s13104-019-4056-z)
Supplement: Supplementary file 6 — Additional file 6: Funnel plot for meta-analysis of the perinatal mortality publication bias among mothers who utilized MWH compared with non-utilized ones. [file 13104_2019_4056_MOESM6_ESM.docx]

Funnel plot for meta-analysis of the perinatal mortality publication bias among mothers who utilized MWH compared with non-utilized ones.

 The estimated bias coefficient (intercept) is 6.1with a standard error of 2.89, giving a p-value of 0.067. The test thus provides strong evidence for the absence of small-study effects
